# Gaze differences in configural and elemental evaluation during multi-attribute decision-making

**DOI:** 10.3389/fnins.2023.1167095

**Published:** 2023-08-25

**Authors:** Juliette Ryan-Lortie, Gabriel Pelletier, Matthew Pilgrim, Lesley K. Fellows

**Affiliations:** ^1^Department of Neurology and Neurosurgery, Montreal Neurological Institute, McGill University, Montreal, QC, Canada; ^2^Department of Psychology, University of Southern California, Los Angeles, CA, United States

**Keywords:** neuroeconomics, sequential sampling models, attention, eye-tracking, value

## Abstract

**Introduction:**

While many everyday choices are between multi-attribute options, how attribute values are integrated to allow such choices remains unclear. Recent findings suggest a distinction between elemental (attribute-by-attribute) and configural (holistic) evaluation of multi-attribute options, with different neural substrates. Here, we asked if there are behavioral or gaze pattern differences between these putatively distinct modes of multi-attribute decision-making.

**Methods:**

Thirty-nine healthy men and women learned the monetary values of novel multi-attribute pseudo-objects (fribbles) and then made choices between pairs of these objects while eye movements were tracked. Value was associated with individual attributes in the elemental condition, and with unique combinations of attributes in the configural condition. Choice, reaction time, gaze fixation time on options and individual attributes, and within- and between-option gaze transitions were recorded.

**Results:**

There were systematic behavioral differences between elemental and configural conditions. Elemental trials had longer reaction times and more between-option transitions, while configural trials had more within-option transitions. The effect of last fixation on choice was more pronounced in the configural condition.

**Discussion:**

We observed differences in gaze patterns and the influence of last fixation location on choice in multi-attribute value-based choices depending on how value is associated with those attributes. This adds support for the claim that multi-attribute option values may emerge either elementally or holistically, reminiscent of similar distinctions in multi-attribute object recognition. This may be important to consider in neuroeconomics research that involve visually-presented complex objects.

## 1. Introduction

Whether choosing your major in college or what to eat for breakfast, decisions are often between complex options with multiple value-predictive attributes. At breakfast, for example, options might be evaluated on their healthiness, taste, and visual appeal. How are these multiple attributes considered during decision-making? In principle, multi-attribute options might be evaluated “elementally” by aggregating the subjective motivational value of individual attributes, or “configurally” by assigning a value to the whole option based on its unique combination of attributes.

Research on multi-attribute decision-making has generally studied elemental evaluation strategies, often using process tracing methods (Ford et al., 1989). Attributes are typically explicitly presented in tables (a.k.a. information boards), usually as text, and the pattern of information acquisition as participants view each piece of information is used to shed light on the underlying evaluation processes (Payne, 1976; Payne et al., 1992; Bettman et al., 1998). Other research on value-based decision making has used visually presented multi-attribute objects such as foods, trinkets, or artwork either without addressing how overall values emerge from these attributes (Busemeyer et al., 2019), or by assuming that individual elements are valued and then combined to estimate the overall value or influence choice (Lim et al., 2013; Suzuki et al., 2017; Vaidya et al., 2018).

An alternative perspective on multi-attribute evaluation is offered by neuroscience research on complex object recognition. This work has established that there are distinct neural substrates for representations of lower-level visual features and for multi-feature configurations that allow whole object recognition (Riesenhuber and Poggio, 1999; McTighe et al., 2010). For example, perirhinal cortex damage impairs object recognition based on unique configurations of features while sparing ‘elemental’ recognition based on individual features (Bussey et al., 2005). There are also behavioral differences suggesting that information is acquired differently in these two forms of object recognition: for example, gaze patterns differ during configural compared to elemental recognition of faces (Bombari et al., 2009, 2013; Boutet et al., 2017).

Similar elemental and configural distinctions recently have been proposed for the evaluation of multi-attribute objects. Damage to ventromedial frontal lobe (VMF), a region implicated in tracking option value, was associated with impaired choices between novel multi-attribute pseudo-objects (fribbles) only when value was related to attribute configuration; choices based on summing the individual values of attributes remained intact (Pelletier and Fellows, 2019). A follow-up eye-tracking and fMRI experiment that asked healthy people to estimate the value of fribbles presented one at a time found that gaze patterns, as well as activity in VMF and perirhinal cortex, differed in configural and elemental value conditions (Pelletier et al., 2021).

The decision neuroscience literature has argued that gaze, as a proxy of attention, can provide additional insights into choice processes beyond what may be inferred from reaction time and choice behavior (Krajbich et al., 2010). Binary value-based choice experiments have shown that longer fixation of an option increases the likelihood of choosing that option, that the option that is fixated last has a higher probability of being chosen, and that the option fixated more over the course of a trial is more likely to be chosen (Krajbich, 2019), in line with models proposing sequential sampling of information to reach a decision. Of note, such tasks have typically involved visual images of complex multi-attribute objects such as snack foods or trinkets.

Here, we asked if these behavioral and eye-tracking outcomes differ across configural and elemental multi-attribute evaluation conditions in healthy young men and women. Participants made binary value-based choices between fribble stimuli in two conditions, with value either associated with individual attributes or the configural relationship between attributes. Because fribble attributes are spatially distinct, eye-tracking could be used to infer information acquisition strategies at both the attribute and the whole option level. We hypothesized that gaze transition patterns would systematically differ across conditions, with the elemental condition prompting an attribute-based strategy with more between-object transitions and the configural condition promoting option-based information acquisition with more within-object transitions. We also tested whether the relationships between gaze patterns and choice considered to be hallmarks of sequential sampling differed across conditions.

## 2. Materials and methods

Forty-one adults were recruited from the local community through online advertising. Participants had normal uncorrected vision, no history of neurological or psychiatric conditions, and had no prior experience with the fribble stimuli. In addition to compensation of 15$ per hour, participants received a monetary bonus based on task performance. Two participants did not complete the full experiment due to time constraints. The final sample of 39 participants had a mean age of 22y, SD 2.7, mean education 16y, SD 2.1, and was comprised of 25 women and 14 men. Participants gave written informed consent in accordance with the Declaration of Helsinki. The study was approved by the McGill University Health Centre Research Ethics Board.

All participants were tested in-lab on a desktop computer equipped with a 19-inch monitor. Experiments were programmed in Matlab (version 2019b, The Mathworks, Inc.), using the Psychtoolbox extension (Brainard, 1997). Eye movements were recorded from the left eye with a desk-mounted eye tracker (EyeLink 1,000 Plus, SR Research) with a sampling frequency of 1,000 Hz. Fixation information was extracted from the EyeLink algorithm (position, duration). To separate attributes of fribbles into distinct regions of interest (ROI), we partitioned the fribbles in two using a Voronoi tessellation. A fixation within an ROI of a fribble followed by a fixation in the other ROI of the same fribble was coded as a within-object transition, and a fixation within an ROI of a fribble followed by a fixation in an ROI of the other fribble was coded as a between-object transition.

The experimental paradigm was adapted from Pelletier and Fellows (2019). Stimuli were renderings of three-dimensional pseudo-objects called fribbles, originally developed for object processing research (Williams and Simons, 2000; Barry et al., 2014). Fribbles are composed of one main ‘body’ and several appendages, which we refer to as attributes. Each set of fribbles had three possible forms for the upper attribute (A, B or C) and the lower attribute (x, y or z). Participants were told that these fribbles were collectors’ items, and that they would learn their market values by observing online ‘auctions’. In the learning phase of the task, they were trained on the approximate monetary values of each fribble by watching such auctions until they reached a learning criterion. This was followed by a choice phase, where participants chose between pairs of fribbles while eye-tracking data were acquired. Chosen fribbles were added to the participant’s own inventory. Participants were told they would sell their inventory back to the experimenter at the end of the experiment, and receive a monetary amount proportional to the proceeds of the sale as part of their compensation for participation, thus incentivizing them to choose the highest value fribbles throughout the choice phase.

**Figure 1:**
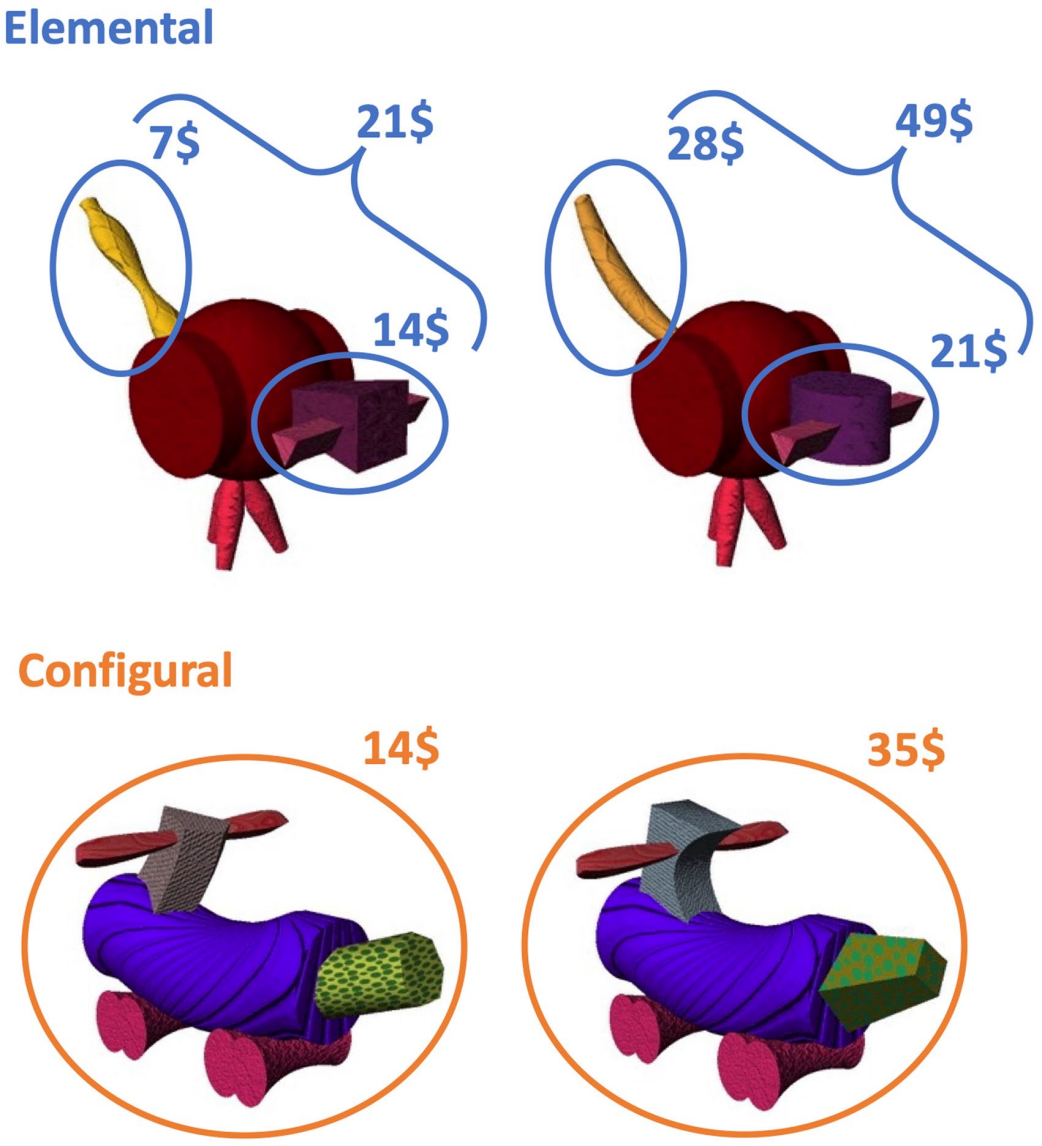
Stimuli. Example of pairs from two families of fribbles, used for each condition. In the elemental condition, each attribute has a value, and a fribble’s value is the sum of its two attributes’ values. In the configural condition, no value is assigned to attributes individually, rather each attribute combination has a value.

The task had two conditions: in the configural condition, the value of each fribble was predicted by the object as a whole through the configural relationship of two attributes (i.e., unique attribute combinations, denoted here Ax, By, Cz, Cx, Bz, Ay). This meant that an individual attribute did not predict a unique value; instead, its value could only be inferred in the presence of the second attribute, i.e., the value was related to the whole object. In the elemental condition, each individual attribute (denoted here A, B, C and x, y, z) was associated with a unique value, i.e., the value of one attribute did not depend on the other attributes that made up the fribble. Thus, the overall fribble value could be derived by summing individual attribute-values (see Figure 1). Different sets of fribbles were used for each condition, counterbalanced across participants. In the elemental condition, all possible combination of attributes were shown, resulting in 9 fribbles. Six unique fribbles were used in the configural condition.

**Figure 2:**
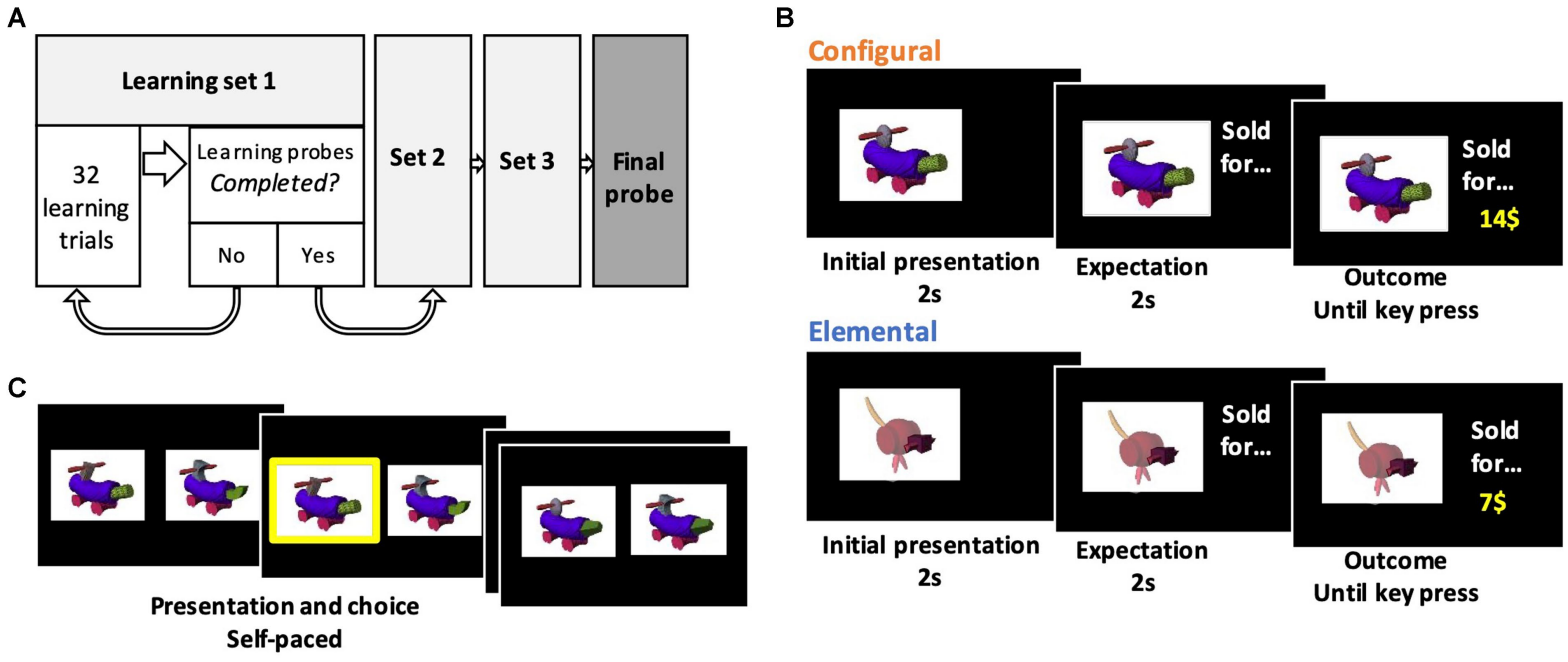
Experiment paradigm. **(A)** Structure of the experiment. Participants went through 3 cycles of learning trials and probes with a subset of stimuli to learn the values of either attributes (in the elemental condition) or configurations of attributes (in the configural condition). **(B)** Learning trials. These trials included presentation of a fribble (with a mask highlighting a specific attribute in the elemental condition), followed by an associated monetary amount. **(C)** Probe trials. These trials had two fribbles presented and participants were instructed to pick the higher-value fribble using arrows on the keyboard in a self-paced fashion. In the elemental learning probes, specific attributes were highlighted like in the elemental learning trials depicted in panel **B**.

The structure of the experiment is depicted in Figure 2A. In the elemental condition, participants were trained on the value associated with individual attributes. To simplify the training, in each learning set only 4 out of the 6 possible attributes were used (e.g., *A*, *B*, x, *y*). On each training trial, one fribble was shown, and the selling price then appeared in the upper right corner of the screen (Figure 2B). Monetary values were drawn at random from a distribution around the following mean values: A = 7$, B = 28$, C = 42$%; x = 14$, y = 21$, z = 35$. Learning trials were self-paced. Participants were instructed that the selling price was related to a single attribute of the fribble. This instruction was further emphasized by highlighting the informative attribute, i.e., by masking the body and irrelevant attributes of the fribble with a semitransparent mask. Each fribble was shown 8 times, for a total of 32 learning trials in a block, followed by a learning probe. The learning probe trials showed two fribbles on the screen, varying on the same 4 attributes as during training, with the relevant attribute highlighted as during training, and participants were instructed to select the fribble whose “highlighted” attribute had the higher value, using left and right arrow keys (Figure 2C). In the learning probe, the 6 possible pairs were shown 8 times, for a total of 48 learning probe trials. The learning probe was stopped if the participant made the same mistake (chose the lower-value attribute) more than once. In this case, the learning block was repeated. The next learning set used another subset of 4 attributes out of the possible 6 (ex: *B, C, y* and *z*), as did the third learning set (ex: *C, A, z* and *x*), following the same training.

After completing the three learning sets, participants then moved to the final probe, involving all 6 attributes presented in all possible pairs (9 fribbles). Each of the 36 possible choice combinations were presented 5 times for a total of 180 choice trials. On these trials, participants were told to consider the values of both attributes of each fribble when making their choice. No mask was used on these trials, so all attributes were equally visually salient. These trials were self-paced. Participants’ gaze was tracked during these final probe trials.

Training in the configural condition followed the same format as the elemental condition except that no mask was used during learning and there were only 6 fribbles. Participants were instructed that selling price was related to the unique combination of attributes that made up the fribble as a whole, and were trained on a subset of 4 fribbles at time until they met the learning criterion. The selling values were drawn at random from a distribution around the following mean values: Ax = 7$, By = 14$, Cz = 21$, Cx = 28, Bz = 35, Ay = 42. This was followed by a final probe in which each of the 15 possible combinations of attributes was shown 6 times for a total of 90 trials.

The primary analysis included all 90 configural trials and a subset (*N* = 77) of the elemental trials that were matched to the configural trials for decision difficulty in terms of relative value and the requirement to consider both attributes of each fribble. This meant that choices in which the value difference between fribble pairs was greater than the range of value differences in the configural set (maximum difference $35), or where fribbles only differed by a single attribute, or where a single attribute was of such high value that on its own it was greater than the sum of the values of the attributes of the other fribble, were excluded.

Linear mixed effect (LME) and generalized linear mixed effect (GLME) models were implemented in R (lme4 package, Bates et al., 2015). LME models were used when the dependent variable was continuous. GLME models were used when the dependent variable was either a count or a binomial, and were fitted with either a Poisson or a binomial distribution, respectively. All models included subject as a random factor, and other predictors are specified for each model in the Results section. Model outputs of LMEs are estimates and are interpretable directly. GLME models for count data provide incidence rate ratios (IRR). Here, these are interpretable as the occurrence ratio of the dependent variable in the configural condition compared to the elemental condition. Since the elemental condition was the reference condition, an IRR > 1 indicates a higher rate of occurrence in the configural condition, and an IRR < 1 indicates a higher rate of occurrence in the elemental condition. GLME models for binomial data output odds ratios. These ratios indicate, as a proportion, the relative occurrence of the dependent variable for the configural condition compared to the elemental condition. The elemental condition was the reference condition, so an odds ratio > 1 indicates a higher occurrence in the configural condition while an odds ratio < 1 indicates a lower occurrence in the configural condition. For GLME models with 2-way interactions, odds were calculated according to the following formula:

odds=EXP(E+C∗c+F∗f+I∗c∗f)

where *E* = intercept estimate, *C* = condition estimate, *c* = condition code (0 for elemental and 1 for configural), *F* = other fixed factor estimate, *f* = other fixed factors code (or value if continuous), and I = interaction estimate.

Payne Index values were calculated from the eye-tracking data for each trial with the following formula:

$$PI=\frac{\left(transitionswithinobjects-transitionsbetweenobjects\right)}{\left(transitionswithinobjects+transitionsbetweenobjects\right)}$$

The value can range between −1 to +1, with −1 indicating an entirely between-object strategy and + 1 indicating an entirely within-object strategy.

## 3. Results

### 3.1. Learning

The number of learning blocks required to meet criterion is shown in Table 1. Participants required more training to meet criterion in the configural than the elemental condition. In the configural condition, 67% of learning sets were learned after one training block and 21% required two training blocks, compared to the elemental condition where 91% of sets were learned after one training block and 8.6% required two blocks. A chi-square test of independence showed that there was a significant association between condition and learning (*X*^2^ (3, *N* = 234) = 25.31, *p* < 0.001, φ = 0.33).

**Table 1:** Behavioral outcomes by condition: the first row shows performance during the training phase; all other outcomes are from the final probe phase of the experiment.

	Elemental condition		Configural condition	
	Mean	SD	Mean	SD
Learning (# of blocks per training set)	1.09	0.17	1.48	0.52
Accuracy (% correct)	0.95	0.23	0.89	0.31
Reaction time (ms)	3,817	4,497	3,155	2,975
Number of attributes fixated per trial	3.57	0.07	3.48	0.05
Within-object transitions per trial (count)	3.85	3.58	3.81	3.58
Between-object transitions per trial (count)	1.95	2.23	1.33	2.23
Payne Index w-b/(w + b)	0.27	0.54	0.43	0.5
Trials where last fixated option was chosen (%)	68%	0.47	76%	0.47
Trials where most fixated option was chosen (%)	65%	0.48	64%	0.48
Proportion of gaze time spent on chosen option (%)	56%	0.15	57%	0.17

### 3.2. Reaction time and accuracy

We first verified that elemental trials where a single attribute was sufficient to make the correct choice (where fribbles only differed on a single attribute or where there was a ‘dominant’ attribute that alone was of higher value than any combination of attributes) were behaviorally distinct from the two-attribute elemental trials of interest. The reaction times were indeed much shorter for the elemental trials involving fribbles that differed on a single attribute (M 2697 ms, SD 2101) compared to the elemental trials where both attributes differed (M 3817 ms, SD 4497; *t* (5380) = −11.2, *p* < 0.001, *d* = 0.86) and to configural trials (M 3155 ms, SD 2975; t(5887) = −6.51, *p* < 0.001, d = 0.37). All remaining analyses focused on the elemental trials that required consideration of two attributes for each fribble, as this was most comparable to the information processing requirements of the configural condition.

An optimal response was defined as the choice of the objectively higher value fribble. Although the difficulty of the decisions based on the subjective value difference between options was similar across conditions, and participants were trained to the same criterion in both conditions, choices were slower and more accurate in the elemental condition (Table 1). GLME models with condition as a predictor confirmed a significant effect of condition on proportion of optimal choices, and on and reaction time (Table 2).

**Table 2:** Reaction time and proportion of optimal choices.

Reaction time		Estimate	CI	*p*-value
*Elemental vs configural*	**Intercept**	3.820	[3.36, 4.27]	<0.001
	**Condition**	−0.662	[−0.83, −0.49]	<0.001

Optimal choices made		Odds ratio	CI	*p*-value
*Elemental vs configural*	**Intercept**	21.81	[16.29, 29.21]	<0.001
	**Condition**	0.47	[0.39, 0.57]	<0.001

### 3.3. Eye-tracking indicators of information acquisition

Eye-tracking data indicated that all four informative attributes were fixated on most trials in both conditions, with a mean of 3.57 attributes fixated per trial in the elemental condition and 3.48 in the configural condition (Table 1). A GLME model analysis with condition as a predictor revealed a marginal effect of condition (*p* = 0.069), with participants tending to fixate more attributes in the elemental than in the configural condition.

**Figure 3:**
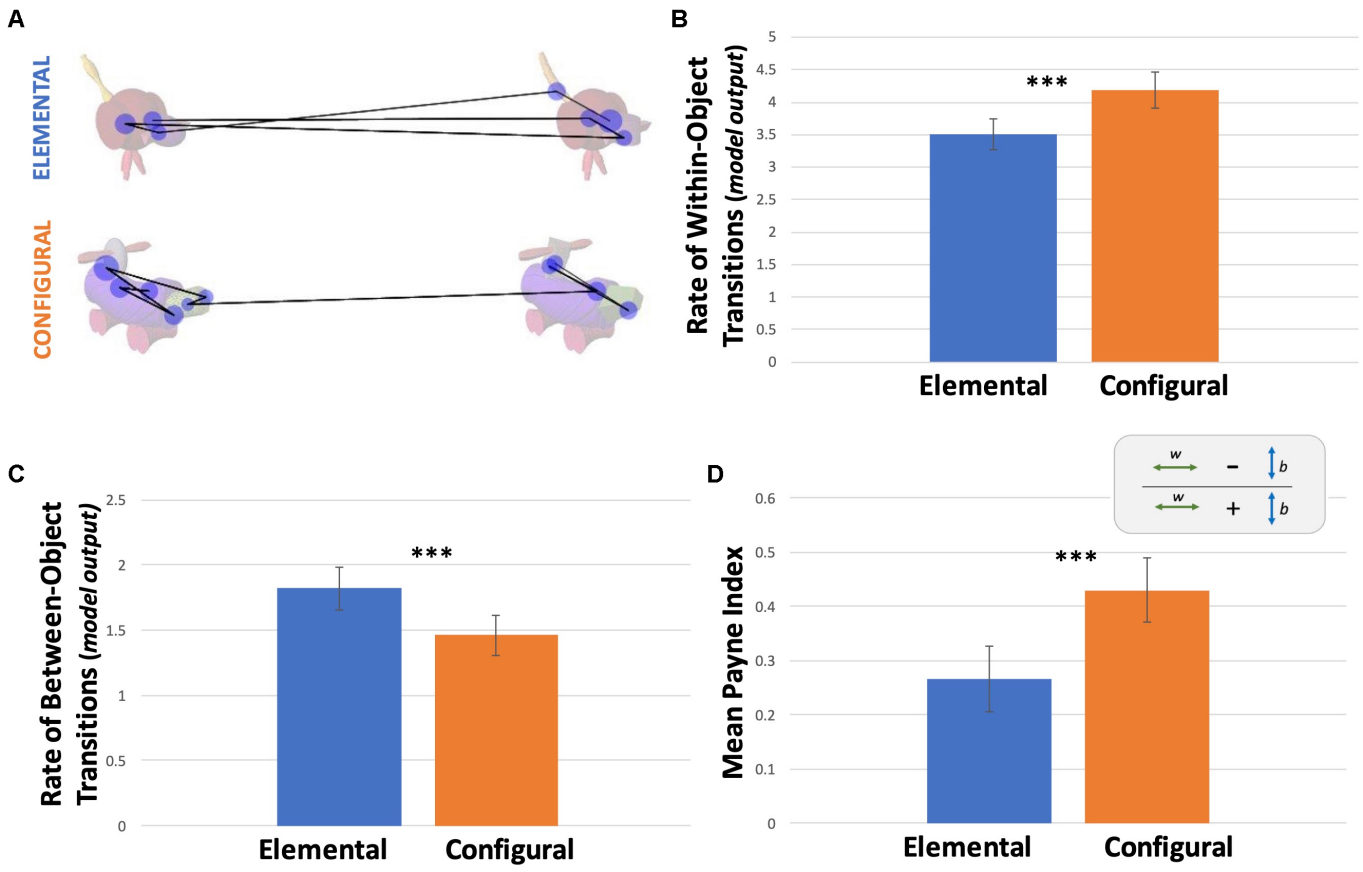
Information Acquisition Patterns. **(A)** Examples of scan paths for both conditions from Subject 216. Lines represent transitions and blue circles represent fixations. **(B)** Estimated rates from the model for within-object transitions across conditions (model output). **(C)** Estimates rates from the model for between-object transitions across conditions (model output). Estimated rates in **B** and **C** account for trial duration differences between conditions. **(D)** Average Payne Index for both conditions calculated from raw number of within (w) and between (b) transitions, P = (w-b)/(w + b). Error bars represent 95% confidence intervals.

We next compared gaze transition metrics, taking an approach inspired by process tracking in decision psychology “information board” experiments. Raw counts of gaze transitions within objects and between objects were compared across conditions (Table 1). A GLME model fitted with a Poisson distribution with condition as a predictor revealed no significant effect of condition on the raw count of within-object transitions (*p* = 0.351; Table 3). The same model on the raw count of between-object transitions yielded a significant effect of condition with more between-object transitions in the elemental (*M* = 1.95) than in the configural condition (M = 1.33). The IRR indicates that the model predicts more between-object transitions in the elemental condition than the configural condition with a ratio of 1 to 0.68 (*p* < 0.001; Table 3). This difference in between-object transitions persisted even after accounting for the observation that trials were longer in the elemental condition, by adding RT at the trial level as an offset variable predictor. Accounting for RT also revealed a significant effect of condition on within-object transitions (Table 3; Figure 3).

**Table 3:** Model output for effects of condition on within-object and between-object gaze transitions.

		Incidence rate ratio	CI	*p*-value
Raw counts				
*Within-object transitions*	**Intercept**	3.59	[3.17, 4.06]	<0.001
	**Condition**	0.99	[0.96, 1.01]	0.351
*Between-object transitions*	**Intercept**	1.83	[1.64, 2.05]	<0.001
	**Condition**	0.68	[0.66, 0.71]	<0.001
Adjusted for RT				
*Within-object transitions*	**Intercept**	1.01	[0.95, 1.08]	0.701
	**Condition**	1.19	[1.16, 1.22]	<0.001
*Between-object transitions*	**Intercept**	0.53	[0.48, 0.58]	<0.001
	**Condition**	0.80	[0.77, 0.84]	<0.001

Payne index		Estimate	CI	*p*-value
	**Intercept**	0.26	[0.20, 0.32]	<0.001
	**Condition**	0.17	[0.14, 0.19]	<0.001

To allow comparison with the decision psychology literature, we also calculated the Payne Index for each trial across the two conditions to assess the relative occurrence of within-object and between- object transitions. The average Payne Index in the configural condition was 0.43 and 0.27 in the elemental condition (Figure 3D; Table 1). An LME model with condition as a predictor revealed a significant effect of condition (Table 3). Thus, while both conditions tended towards within-object processing (Payne Index >1), this tendency was more evident in the configural condition. Adjusting for reaction time was not necessary, as the difference between the two types of transitions was divided by the total number of transitions for each trial.

### 3.4. Effect of last fixation location on choice

**Figure 4:**
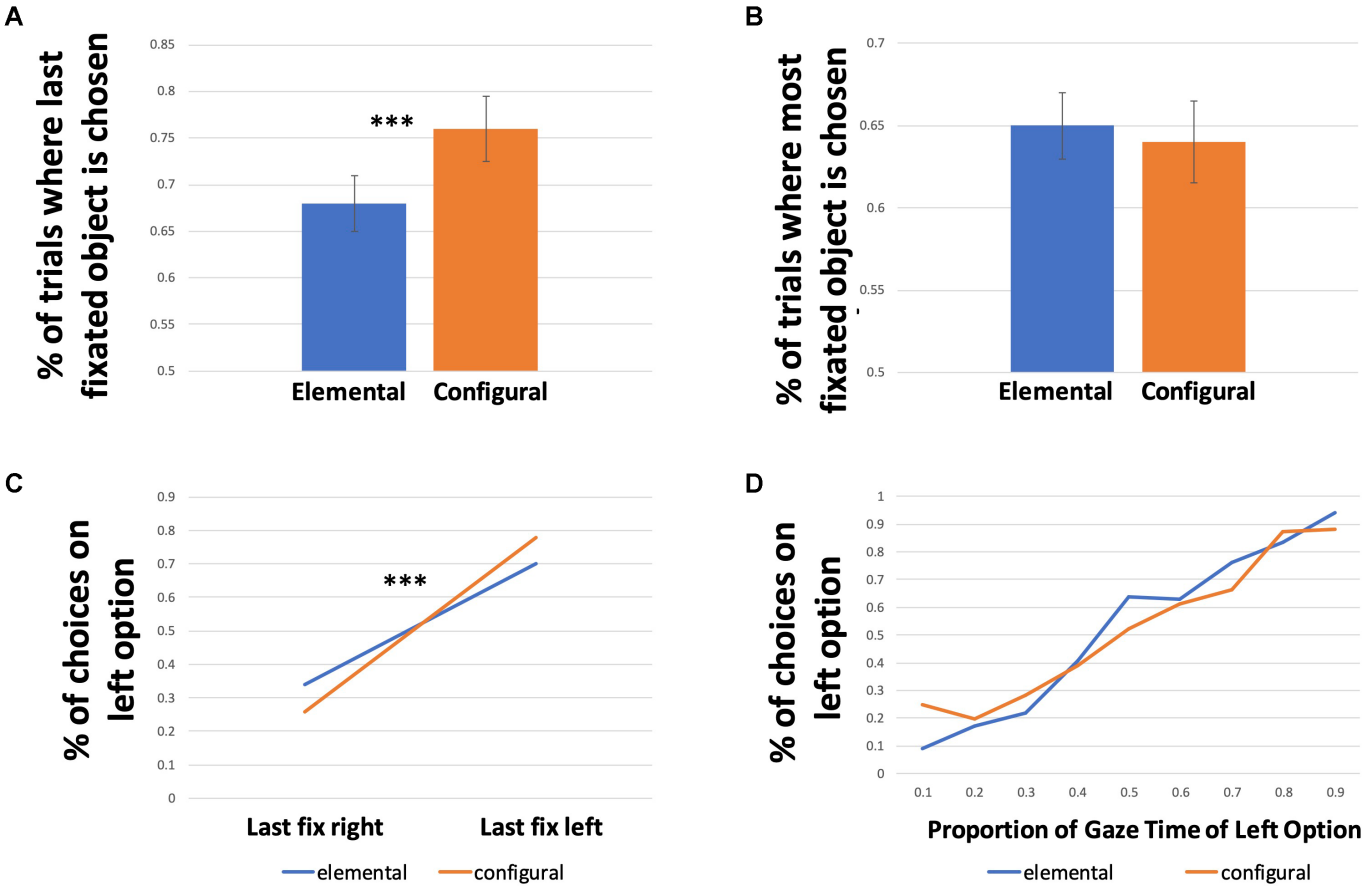
Effects of last fixation and gaze time on choice. **(A)** Proportion of trials where the option that was fixated last was chosen, for both conditions. Error bars represent 95% confidence intervals. **(B)** Proportion of trials where the option with the higher gaze time was chosen, for both conditions. Error bars represent 95% confidence intervals. **(C)** Proportion of trials where the left option was chosen as a function of location of the last fixation, across conditions. **(D)** Proportion of trials where the left option was chosen as a function of gaze time proportion on the left option, across conditions.

We next examined gaze effects on choice predicted by evidence accumulation models. A one-sample *t*-test revealed that for all conditions together, the mean proportion of trials where the last fixated option was chosen (72% of trials, SD 0.45) was significantly different than 50% (*t* (6512) = 40.22, *p* < 0.001, *d* = 0.45). A GLME model with condition as a predictor revealed a significant effect of condition (*p* < 0.001), with more trials where the last fixated option was chosen in the configural condition than in the elemental condition (Figure 4A; Table 1). The Odds Ratio indicates that in the configural condition there were 50% more trials in which the last-fixated option was chosen (Table 4).

**Table 4:** Model output for effect of last fixation on choice.

		Odds ratio	CI	*p*-value
Last fixation chosen	**Intercept**	2.22	[1.88, 2.62]	<0.001
*Proportion of trials*	**Condition**	1.50	[1.34, 1.68]	<0.001

		Estimate	Std. Err.	*p*-value
Effect of last fixation	**Intercept**	−0.67	0.06	<0.001
	**Condition**	−0.39	0.08	<0.001
	**Last fixation**	1.52	0.08	<0.001
	Condition * Last fixation	0.79	0.11	<0.001

Given the apparent difference in effects between conditions, we examined the interaction between last fixation location and condition. The proportion of choices of the left option as a function of whether the left option was fixated last are presented in Figure 4B. In the configural condition, the left option was chosen in 26% (SD 0.44) of the trials where the last fixation was on the right option compared to 78% (SD 0.42) of trials where the last fixation was on the left option. In the elemental condition, the left option was chosen in 34% (SD 0.47) of the trials where the last fixation was on the right option compared to 70% (SD 0.46) of trials where the last fixation was on the left option. A GLME model fit with a binomial distribution with the interaction of condition and last fixation location as a predictor revealed a significant interaction of condition and last fixation location on choice, suggesting that the effect of last fixation location on choice was greater in the configural condition than the elemental condition (Table 4). To estimate the effect size of this difference, we calculated the expected values of the dependent variable, i.e., the proportion of choices, from the regression table output of the model, and exponentiated them to get the odds for each fixed factor level (Table 5). The odds ratio was calculated by dividing the odds of the configural condition by the odds of the elemental condition for each level of last fixation location. For example, the odds ratio of 1.49 for trials where the left option was fixated last indicates that the estimated likelihood of choosing the left option was 49% higher in the configural condition than in the elemental condition.

**Table 5:** Odds and odds ratios for effect of last fixation location.

	Elemental	Configural	Odds ratio
Last fixation on right	0.51	0.35	0.68
Last fixation on left	2.34	3.50	1.49

### 3.5. Effect of gaze time on choice

We asked whether spending a larger proportion of time fixating one option in a trial led to a higher probability of choosing that option. We first assessed whether this effect was present in the data overall, and then whether this differed across conditions. A one-sample t-test revealed that, collapsed across conditions, the mean proportion of trials where the most fixated option was chosen (64% of trials, SD 0.48) was significantly different from 50% (t(6512) = 23.70, *p* < 0.001, d = 0.29), suggesting an overall effect of gaze time advantage on choice. Comparing conditions, we observed that the most fixated object was chosen in 64% of trials in the configural condition compared to 65% of trials in the elemental condition (Figure 4C; Table 1). A one-way GLME model fit with a binomial distribution with condition as a predictor revealed no significant difference between the conditions for the effect of gaze time on choice (Table 6). Figure 4D depicts the proportion of left choices as a function of gaze time on the left option for both conditions.

**Table 6:** Model output for effect of gaze time on choice.

		Odds ratio	CI	*p*-value
*Most fixated chosen*				
*Proportion of trials*	**Intercept**	1.85	[1.76, 2.04]	<0.001
	**Condition**	0.95	[0.86, 1.05]	0.311
*Gaze on chosen option*				
*Proportion gaze of time*	**Intercept**	0.56	[0.55, 0.57]	<0.001
	**Condition**	0.001	[−0.00, 0.01]	0.111

We also considered the average proportion of gaze time spent on the chosen object. A one-sample t-test revealed that the overall group mean (56% of gaze, SD 0.16) was significantly different from 50% (*t* (6512) = 31.23, *p* < 0.001, d = 0.39). A one-way LME with condition as a predictor model found no significant difference between gaze time on the chosen object across conditions (Table 1 for means, Table 6 for model output).

## 4. Discussion

This study sought evidence of behavioral differences between configural and elemental multi-attribute option evaluation in a value-based binary choice task between complex visual objects with spatially distinct attributes. There were several behavioral differences identified, largely in line with our hypotheses. Configural evaluation was faster, less accurate, involved more within-option gaze transitions, and there was a greater influence of last fixation on choice. There were also interesting commonalities across conditions. Participants fixated between 3 and 4 value-informative attributes in both conditions and, despite the differences in gaze fixation transitions, the chosen option was overall fixated more than the non-chosen option to a similar degree across conditions.

Process tracing research has used eye movements as indicators of information acquisition during value-based decision-making in multi-attribute choice, but in contexts where option attributes (elements) are described with text, generally arrayed in a table (Russo and Rosen, 1975; Payne, 1976; Raaij, 1977; Payne et al., 1993; Russo and Leclerc, 1994; Russo and Dosher, 1983). The complex object stimuli used here had spatially distinct attributes, allowing both option- and attribute-level fixations to be studied. Our findings show that the process-tracing analysis framework can be applied to complex visual objects. While option-based information acquisition patterns predominated in both conditions (i.e., the Payne Index was on average, positive), this option-based pattern was more marked in the configural condition. The effects observed are in line with our conceptualization of configural evaluation as more option-based, and, elemental evaluation relying on attribute-by-attribute comparisons between options. The spatial proximity of attributes of the same fribble might explain the overall bias towards within-option information acquisition (Russo and Rosen, 1975; Ballard et al., 1995); future work could test this speculation by adapting the fribble stimuli to more widely space the value-informative attributes within-object.

Our findings suggest that the extent to which attributes interact in predicting value is an important factor influencing information acquisition in the service of choice “strategies” (which, here, may or may not be explicit, top-down strategies), revealed by gaze patterns. A study from our lab using a similar paradigm also found systematic gaze pattern differences between elemental and configural conditions when fribbles were evaluated one at a time (Pelletier et al., 2020).

This application of eye-tracking to distinguish configural and elemental evaluation complements perceptual studies where this method has been applied to compare configural and elemental face recognition processes (Bombari et al., 2009, 2013; Boutet et al., 2017). Bombari et al. (2009) found more transitions (and therefore, more fixations) within face stimuli under configural conditions. The authors suggested that different recognition strategies were at play, with the configural condition involving a more pronounced analysis of spatial relations between attributes.

As reviewed in Pelletier and Fellows (2021), there is evidence that value emerges as part of the recognition process rather than through a separate evaluation step that follows recognition (Mogami and Tanaka, 2006; Serences, 2008; Arsenault et al., 2013; Persichetti et al., 2015; Kaskan et al., 2017). Multi-attribute value construction may be organized hierarchically in the brain, from evaluation of basic features and attributes to the evaluation of complex conjunctions and objects, in line with the processing stages underlying object perception. Lesion and neuroimaging studies from our lab using a similar paradigm show differences in the brain regions engaged by, and critical for, configural and elemental evaluation (Pelletier and Fellows, 2019; Pelletier et al., 2021).

We speculate that the familiar complex objects (e.g., snack food packages) used in many binary choice studies in decision neuroscience are likely to promote configural evaluation. We wondered if key behavioral effects that have been taken as support for sequential sampling evidence accumulation models might be unique to, or at least especially prominent in, configural evaluation. Such models typically do not address what information is being sampled during evaluation, although, as with the earlier process-tracking literature, there is often an assumption that value-predictive attributes are being sampled (Lim et al., 2011, 2013; Krajbich et al., 2012). Most recent extensions of sequential sampling models applied to value-based decision-making predict that the last-fixated option is most likely to be chosen (Krajbich et al., 2010; Krajbich and Rangel, 2011; Morii and Sakagami, 2015; Smith and Krajbich, 2019; Liu et al., 2020). Our finding that the effect of last fixation on choice was more striking in the configural condition suggests that sequential-sampling models may be more applicable when option value is assessed holistically. The corollary of this observation is that different models may be needed for decisions where individual elements predict value. Recent efforts to extend attentional drift diffusion models to multi-attribute contexts where attribute values are explicitly considered (Yang and Krajbich, 2022) are promising, as they offer a way to account for potentially distinct mechanisms of multi-attribute evaluation. Further research in this direction is needed, to develop models that allow for either configural or elemental evaluation, and to acquire experimental data to test the predictions of such models.

Evidence accumulation models also predict that longer time spent gazing at an option allows more evidence to be accumulated (Armel et al., 2008; Orquin and Loose, 2013), bringing that option closer to the decision threshold and therefore more likely to be chosen. Here, we replicated this effect. In contrast to the effect of last fixation on choice, overall gaze time advantage for the chosen option was of similar magnitude across conditions, despite the differences in how value was related to the stimuli.

This work has limitations. Although we aimed to match the two conditions for difficulty by training to a common criterion across conditions and matching trials on option value difference, differences in learning and accuracy were observed, with the higher value fribble chosen less often and more learning blocks required to learn the values in the configural trials. The range of absolute values for fribbles also differed between conditions. However, the relative value of fribbles within choices, the most important factor in binary choice behavior, Kim and Beck (2020) was similar across conditions. While participants were instructed to ‘add up’ the learned values of individual attributes in the elemental trials, they could have adopted other attribute-based strategies including shortcuts such as a ‘take the best’ single attribute strategy, which could have yielded high accuracy without considering all attribute-values individually in some trials. However, elemental trial reaction times were longer, and fixation number was similar in both conditions, arguing that participants likely gathered all available value information. Finally, by design, the salience of individual attributes was emphasized during the elemental training phase, through both the value association and partial masking of task-irrelevant portions of the fribble. While no masking was used in the final probe phase, it is possible that the masking during training may have enhanced the effect of the attribute-value association alone.

Gaze is known to be an imperfect indicator of attention and thus of information acquisition. Studies have found that subjects are capable of maintaining fixation on one feature while detecting probes in the close periphery (Fluharty et al., 2016), and that attention can be deployed to multiple non-contiguous areas of the visual field without changing the gaze fixation location (Kramer, 1998). A recent study found poor correspondence between instructed strategy use (based on computer simulation of optimal strategies) and actual information acquisition patterns, but this was in a decision task where the best strategy was cognitively very demanding (Takemura et al., 2023). Given the size of the ROIs used here and their spatial proximity, fixation on one attribute could be enough to recognize the whole fribble. However, we observed fixations to, on average, 3.5/4 informative attributes in both conditions, arguing that gaze was a reasonable proxy for attention, and therefore of the underlying choice strategy, in this self-paced, low time pressure, relatively simple task.

While we observed differences between conditions in several behavioral metrics, these behavioral phenomena alone are unlikely to reliably distinguish elemental and configural evaluation in more naturalistic paradigms where other factors might influence gaze and where attributes may not be spatially distinct. At the least, careful experimental control would be needed for the various additional factors known to influence eye movement patterns, such as spatial arrangement of stimuli (Reutskaja et al., 2011) and social context (Peshkovskaya and Myagkov, 2020). Moreover, when values are not experimentally assigned, as they were here, the evaluation processes and strategies engaged can differ across individuals, as well as within individuals as decision difficulty varies (Lee and Cummins, 2004; Day et al., 2009).

Nonetheless, the behavioral effects we observed may be useful in distinguishing between these evaluation modes in future work. Without expressly considering the elemental-configural distinction we studied here, process-tracing experiments often assume some form of elemental attribute integration. Sequential sampling models are generally agnostic as to how the value-predictive attributes of complex objects are combined during choice. We found that some of the behavioral predictions of such models are more strongly supported under configural evaluation conditions. Different models may be more appropriate for decisions where individual elements predict value.

This study adds to a growing literature that aims to more tightly define the processes involved in assessing the value of complex decision options, with the intent to relate these to their underlying neural substrates. A better understanding of the behavioral and brain mechanisms that underlie how humans make complex choices also may help us present multi-attribute information in ways that the human brain is best prepared to consider.

## Data availability statement

The datasets presented in this article are not readily available because Informed consent was not obtained for open data sharing; at the time the data were collected this was not a routine aspect of the ethics process. Requests to access the datasets should be directed to juliette.ryan-lortie@mail.mcgill.ca.

## Ethics statement

The studies involving humans were approved by McGill University Health Center Research Ethics Board. The studies were conducted in accordance with the local legislation and institutional requirements. The participants provided their written informed consent to participate in this study.

## Author contributions

GP and LF designed the study. MP collected the data. JR-L, GP, and LF conceived of the research questions. JR-L and LF analyzed the data and wrote the first draft and carried out the final revisions of the paper. GP advised on the analysis of the data. All authors contributed to the article and approved the submitted version.

## Funding

This work was supported by the Canadian Institutes of Health Research (Grant MOP-11920) and the Natural Sciences and Engineering Research Council of Canada (RGPIN 2016–06066).

## Conflict of interest

The authors declare that the research was conducted in the absence of any commercial or financial relationships that could be construed as a potential conflict of interest.

## Publisher’s note

All claims expressed in this article are solely those of the authors and do not necessarily represent those of their affiliated organizations, or those of the publisher, the editors and the reviewers. Any product that may be evaluated in this article, or claim that may be made by its manufacturer, is not guaranteed or endorsed by the publisher.
